# Spatial-Temporal Characteristics of Multi-Hazard Resilience in Ecologically Fragile Areas of Southwest China: A Case Study in Aba

**DOI:** 10.3390/ijerph191912018

**Published:** 2022-09-22

**Authors:** Ying Zhou, Qihao Su, Yulian Li, Xingwei Li

**Affiliations:** College of Architecture and Urban-Rural Planning, Sichuan Agricultural University, Chengdu 611830, China

**Keywords:** multi-hazards, disaster resilience, spatial-temporal, DROP, ESDA, mountainous areas

## Abstract

Aba’s topography, weather, and climate make it prone to landslides, mudslides, and other natural disasters, which limit economic and social growth. Assessing and improving regional resilience is important to mitigate natural disasters and achieve sustainable development. In this paper, the entropy weight method is used to calculate the resilience of Aba under multi-hazard stress from 2010 to 2018 by combining the existing framework with the disaster resilience of the place (DROP) model. Then spatial-temporal characteristics are analyzed based on the coefficient of variation and exploratory spatial data analysis (ESDA). Finally, partial least squares (PLS) regression is used to identify the key influences on disaster resilience. The results show that (1) the disaster resilience in Aba increased from 2010 to 2018 but dropped in 2013 and 2017 due to large-scale disasters. (2) There are temporal and spatial differences in the level of development in each of the Aba counties. From 2010 to 2016, disaster resilience shows a significant positive spatial association and high-high (HH) aggregation in the east and low-low (LL) aggregation in the west. Then the spatial aggregation weakened after 2017. This paper proposes integrating regional development, strengthening the development level building, and emphasizing disaster management for Aba.

## 1. Introduction

In recent years, humans are being increasingly exposed to natural disasters with the effects of urbanization, global climate change, and population growth [1]. In 2021, Chinese Ministry of Emergency Management released that 107 million people were affected by various natural disasters, with an economic loss of approximately 334.02 billion CNY, resulting in 867 deaths. Disasters seldom happen alone. Multiple recurrent natural catastrophes may cause greater economic damage than one-time calamities [2]. Multiple disasters bring more severe economic and social repercussions on developing countries than on developed ones, potentially causing negative economic development [3]. In this situation, resilience became a global guiding principle for natural hazards. In 2015 the United Nations Office for Disaster Risk Reduction (UNDRR) proposed to monitor, assess, and comprehend disaster risks, strengthen resilience, and make disaster risk reduction actions multi-hazardous [4]. Resilience is a conceptual tool for understanding how people groups respond to disasters that affect their livelihoods. It is also a protective factor, and better resilience often responds to better coping, absorption, and adaptation to the impacts of natural hazards [5]. As a result, one of the main objectives of current disaster prevention and mitigation research is to investigate how to improve resilience. Research on it can provide substantive support for the decision making of disaster resilience and mitigation, society, and the environment. It can serve as inspiration for those involved in development and disaster management. Resilience research also has important implications for regional sustainable development [6,7,8]. Resilience emerged as a top priority for policy and planning initiatives to mitigate disaster risk and was a dominating concept in the disaster management research and policy community for the past several years [9].

China is one of the countries that suffer most from natural disasters. According to the Chinese Ministry of Emergency Management, various natural disasters affected a total of 107 million people and caused direct economic losses of 334.02 billion CNY in 2021. Specifically, Sichuan Province, located southwest of China, is more prone to natural disasters. Aba is a mountainous area located in the western Sichuan Province of China. It is situated in an abrupt topographic zone between the Qinghai–Tibet Plateau and the Sichuan Basin. Strong neotectonic movements and high altitudes frequently cause mountain disasters, such as landslides, debris flows, and ground collapse [10]. In addition, the region’s fragile foundation for agriculture and animal husbandry, as well as inadequate infrastructure, obstructed the region’s ability to withstand natural calamities and limited regional development. Therefore, Aba faces more threats from natural disasters. According to data provided by the Natural Resources Bureau of the Aba Tibetan and Qiang Autonomous Prefecture, there are 5155 geological catastrophe risk areas, endangering more than 17 billion properties and 267,898 persons in 2021. Frequent natural disasters and the potential domino effect of disaster hazards increase the risk [11]. It is a challenge to residents‘ daily life, economic development, and even the sustainable development of the entire Aba social-ecological system. Furthermore, across the areas of Aba, there are apparent disparities in economic development, ecology, social resources, and transportation infrastructure. Faced with various natural disaster incursions, the region’s resilience building is relatively insufficient. Therefore, this paper is to investigate the situation of Aba to improve its resilience under the influence of multi-hazards and provide recommendations for disaster prevention and mitigation in Aba.

Resilience is typically studied from a quantitative or qualitative perspective [12]. Research focused on the following areas:

Definition of the concept of resilience. Currently, there is no academic consensus on the definition of resilience [12,13]. Resilience originated in the field of ecology [14]. Then, the concept was gradually applied to psychology, economics, engineering, urban development, and natural disasters. Due to the emergence of various definitions, such as engineering resilience, economic resilience, ecological resilience, social resilience, livelihood resilience, and disaster resilience [7,15,16], the definition of the concept of resilience became a hot topic. Resilience usually refers to the ability of a system to reorganize and return to its normal working state after a shock [13]. Nonetheless, other researchers consider that resilience prioritizes system stability or is primarily concerned with the speed of recovery once a system is exposed to risk. Resilience is a result, while others view it as a process [17].

Evaluation system and method. Using appropriate evaluation methods and establishing a reasonable indicator evaluation system is a deepening of the concept of resilience [18]. Resilience is measured by the amount of disturbance a system can absorb while maintaining its original function [19]. However, it is difficult to directly obtain the amount of disturbance, and it is frequently expressed through indicators and various dimensions and scales. As for dimensions, some researchers incorporated structural dynamics theory [20], community baseline models [21], projection pursuit clustering (PPC) models [1], and disaster resilience of place (DROP) models [22] to develop indicator systems from multiple dimensions, including infrastructure, environment, economy, society, and institutions. Some researchers also established indicator systems for single dimensions of disasters, such as earthquakes [23], floods [24], and landslides [25]. As for scales, resilience exists at multiple levels; so numerous researchers conducted separate studies for communities [26], urban [27], and regions [28].

The improvement strategies and paths. To apply resilience assessment research to practice, it is required to suggest focused improvement approaches and techniques. Increasing resilience strategy research indicates the progressive use of resilience theory in practice [29]. Some geographical or econometric instruments are frequently used to process measurement results to formulate targeted recommendations. Exploratory spatial data analysis (ESDA) is utilized to analyze the spatial and temporal characteristics of the distribution of resilience indices [18,28]. Using Gini coefficients [30] and coefficients of variation [31], cities’ and regions’ levels of development and differences in their resilience are analyzed. There are statistical relationships between the independent and dependent variables, such as covariance, factorial interactions among variables, etc. Therefore, researchers used many methods to identify key influences based on the statistical relationships between indicator variables. For example, the geographical detectors (no linearity assumption), ordinary linear regression (no multiple covariances of indicators), partial least squares regression (multiple covariances between indicators), and ridge regression (multiple covariances between indicators) [28,32,33].

In summary, researchers conducted various studies on resilience and achieved many results, but some issues still need to be explored in depth.

(1) Currently, studies mainly focus on specific dimensions of urban resilience, such as economic, social, ecological, or infrastructural resilience [29]. Urban areas and communities are frequently exposed to multiple hazards, increasing their vulnerability [11]. However, most studies only address single-dimensional natural hazards, such as earthquakes, floods, and landslides, and few examine the effects of multi-hazards [34].

(2) Different evaluation systems and models apply to different areas, so there are different evaluation methods for coastal cities, inland areas, and highland mountainous areas [35]. Meanwhile, the indicators chosen for resilience may vary for different disasters [12]. Therefore, a variety of factors, including the capability approach based on copying, adaptive, and transformative capacity [36], as well as the selection of social, economic, community, neighborhood, infrastructure, environmental, spatial, and management dimensions [37,38], are all used by researchers to measure resilience. Consequently, the existing framework models must be adapted to different circumstances to measure the resilience of multiple hazards.

(3) Most studies focus on enhancing urban areas’ resilience while ignoring rural areas’ resilience. Due to their inaccessibility and marginalization, mountainous regions are vulnerable [39,40]. In mountainous areas with low population density, weak institutional capacity, and a limited economy, how to effectively mitigate the harmful effects of multiple natural hazards and strengthen regional resilience became a vital issue for sustainable development [41].

Considering that there are few studies on mountainous areas under the influence of multi-hazards, this paper studies Aba, a highland mountainous region with frequent geological hazards, as the research object. An indicator evaluation system is constructed by the three parts of disaster prevention, resistance and rescue, and the indicator weights are determined using the entropy weighting method to calculate the multi-hazard disaster resilience of landslide, debris flow, and collapse from 2010 to 2018. The study combines exploratory spatial data analysis (ESDA) and multiple linear regression to explore the spatial-temporal differences and influencing factors of disaster resilience. It should be noted that in our study, resilience defines as the ability of a system to use environmental, economic, and social capital to mitigate or resist shocks [13]. Meanwhile, this paper divides Aba into regions according to counties and considers each region as a system to study the resilience of each region. As a result, this paper uses indicators from four dimensions: environmental, economic, infrastructural, and social, in each region to analyze regional resilience.

The contributions of this study to the existing research are as follows: (1) Most of the existing studies focus on urban areas, but in our study, highland mountainous areas are selected as the study unit with counties. (2) A multi-dimensional study of disaster resilience is considered under the influence of multiple disasters, such as debris flows, landslides, and collapses. (3) This paper studies the factors influencing multi-hazard disaster resilience with multiple linear regression, which can provide a reference for formulating disaster management and regional development strategies to achieve sustainable and synergistic regional development.

The remainder of this paper is arranged as follows: Section 2 details the study area and the methods employed to compute disaster resilience. In Section 3, the resiliency calculation results are shown, together with the spatial-temporal analysis of disaster resilience and the identification of key influencing factors. Section 4. We discuss the reasons for the phenomenon of Section 3 and put forward corresponding policy suggestions. The last part of the article is the conclusion.

## 2. Materials and Methods

### 2.1. Research Area

**Figure 1:**
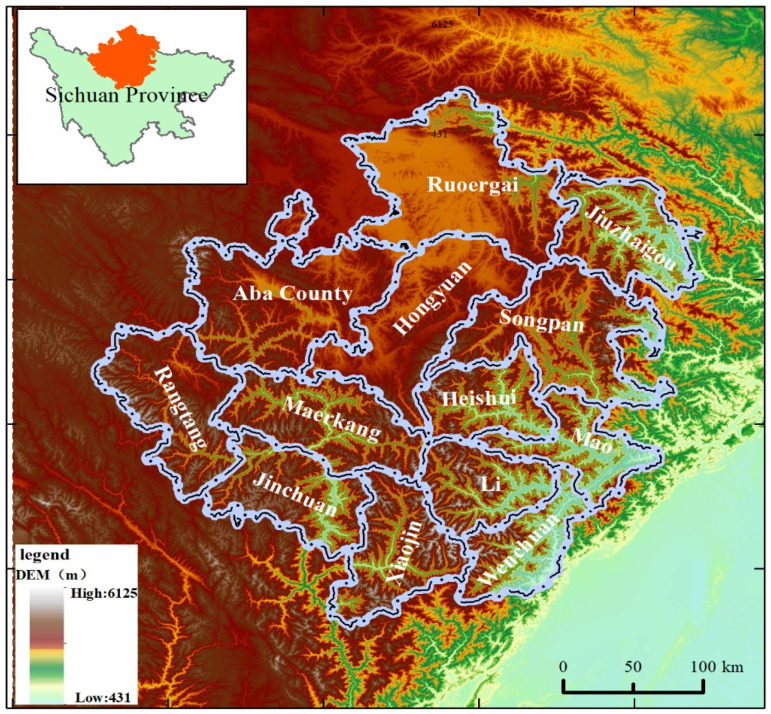
Aba Location Map.

Aba Tibetan and Qiang Autonomous Prefecture in Sichuan Province is located on the southeastern edge of the Qinghai–Tibetan Plateau, between 100°0′~104°7′ E and 30°5′~34°9′ N. It is about 414 km long from north to south, 360 km wide from east to west, covering 84,242 square kilometers, with an average altitude of over 3000 m. The prefecture is a typical plateau with high terrain and comprises 13 districts in Maerkang City and 12 counties, including Jinchuan, Xiaojin, Aba, Ruoergai, Hongyuan, Zaand ngtang, Wenchuan, Lixian, Maoxian, Songpan, Jiuzhaigou, and Heishui. As a mountainous area in western Sichuan, most of the Aba Prefecture belongs to alpine and canyon areas, with high mountains and steep slopes, complex geological structures, broken rock formations, unstable slopes, loose accumulations, high-risk and high-level dangerous rock bodies, debris formed by earthquakes, and weighty rainfall makes it vulnerable to multiple disasters, becoming a high-incidence area for earthquakes, collapses, landslides and debris flow disaster [1]. The location map of Aba is shown in Figure 1.

### 2.2. Data Sources

The socioeconomic data collected in this paper are mainly from *Sichuan Statistical Yearbook* and *Aba Statistical Yearbook* (2010–2018). The *County Statistical Yearbook* for 13 counties, including Maerkang, Jinchuan, Xiaojin, Aba, Ruoergai, Hongyuan, Rangtang, Wenchuan, Li, Mao, Songpan, Jiuzhaigou, and Heishui (2010–2018) were used. The data used to calculate the disaster pressure mainly comes from the Natural Resources Department of Sichuan Province and the people’s governments of various counties.

### 2.3. Measurement Methods

#### 2.3.1. Resilience Calculation

This paper adopts and improves the computational model of Tian (2019), and quantifies the resilience by studying the gap between the ideal and actual functions of the area [41] because disaster resilience refers to a system’s capacity to withstand and lessen shocks in response to disaster disturbances.

Disaster resilience can be expressed as the reciprocal of system losses, which is calculated as follows:

(1) R=1/(I−Q)

(2) $${Q=f}_{r}{\times f}_{p}$$

In Equations (1) and (2), R represents the disaster resilience, I represents the ideal function of the system, Q represents the actual function of the system under the influence of natural disasters, $f_{r}$ represents the function of each dimension of the system, and $f_{p}$ represents the disaster pressure.

This paper assumes that the ideal function of the system I is the maximum value of each dimension in the study area from 2010 to 2018 to calculate the composite score. The direct disaster losses reflect the most direct impact of disasters on the region. However, due to our definition of resilience, the direct disaster loss is indirectly reflected in each dimension of the system, and we only used the disaster hazard points to calculate the disaster pressure.

#### 2.3.2. Global Entropy Weight

The principle of the entropy weight method is to use the discrete degree of the indicators to determine the weight of different indicators. If a class of indicators is more important in the evaluation system, the information entropy is smaller, and the disorder is smaller, so the weight is higher, and vice versa [42].

Since the data in this study are panel data of 13 regions, the global entropy weighting method is used to determine the indicator weights to evaluate disaster resilience in Aba under the influence of multi-hazards. In other words, a three-dimensional time-series data table involving regions, indicators, and time is established for evaluation by introducing time series in the cross-sectional data.

(1) Setting indicators.

Assuming there are *n* evaluation areas, *h* evaluation indicators, and *T* years, the overall evaluation matrix is as follows:

(3) $$X={\left\{x_{ij}^{t}\right\}}_{nT\times h}\;$$

In Equation (3), $x_{ij}^{t}$ represents the *j*^th^ evaluation index of the *i*^th^ area in the *t*^th^ year.

(2) Standardize the original value of indicators.

Since the original dimensions of the indicators are different, it needs to be normalized to a dimensionless value between 0 and 1 to become a comprehensive indicator. At the same time, since there are positive and negative indicators, they need to be dealt with separately. Therefore, the paper adopts the range method to standardize the indicators, and the equation is as follows:

(4) $$Z_{ij}^{t}=\frac{X_{ij}^{t}-X_{ij\;min}}{X_{ij\;max}-X_{ij\;min}}\;$$

(5) $$Z_{ij}^{t}=\frac{X_{ij\;max}-X_{ij}^{t}}{X_{ij\;max}-X_{ij\;min}}$$

In Equations (4) and (5), $X_{ij}^{t}$ is the index value of item *j* in year *t* in the *i*^th^ study area. $X_{ij\;max}$ and $X_{ij\;min}$ are the maximum and minimum values of the screening indicators, respectively. $Z_{ij}^{t}$ is the standardized value of each indicator. Equations (4) and (5) are used for positive and negative indicators.

The higher the average altitude, the more backward socio-economic conditions and the greater the vulnerability to disasters within the study area. Moreover, the number of hidden danger points involved in debris flow, landslide, and collapse in the disaster pressure index represents the degree of disaster impact on the region. The higher the value, the more significant the impact of the disaster on the region. In this paper, except for the indicators involved in the average altitude and the disaster pressure dimension, the other indicators are all positively processed.

(3) Calculate the proportion of indicators

(6) $$P_{ij}^{t}=\frac{Z_{ij}^{t}}{\sum_{t=1}^{T}\sum_{i=1}^{n}Z_{ij}^{t}}\;$$

in Equation (6), $P_{ij}^{t}$ is the proportion of the *i*^th^ evaluation object on the *j*^th^ indicator in the *t*^th^ year.

(4) Calculate the entropy value of the index

(7) $$H_{j}=-K\overset{T}{\underset{t=1}{\sum }}\overset{n}{\underset{i=1}{\sum }}P_{ij}{\times lnP}_{ij}\;$$

in Equation (7), K is the normalization coefficient, ${K=1/\ln(\mathrm{nT}),\;H}_{j}$ is the entropy of the *t*^th^ index, ${K>0,\;H}_{j}\geq 0$.

(5) Calculate the entropy value redundancy

(8) $$D_{j}{=1-H}_{j}.$$

In Equation (8), $D_{j}$ represents the entropy value redundancy of the evaluation index.

(6) Weights and indicator weighted score

(9) $$W_{j}=\frac{D_{j}}{\sum_{j=1}^{n}D_{j}}\;$$

(10) $$f_{k}=\sum Z_{ij}^{t}W_{j}.$$

In Equation (9), $W_{j}$ represents the weight of the evaluation index, and in Equation (10), $f_{k}$ shows the disaster resilience score of the *k*^th^ area, where *k* is the selected evaluation area.

#### 2.3.3. Exploratory Spatial Data Analysis (ESDA)

Exploratory spatial data analysis (ESDA) is a basic statistical method for exploring the characteristics of the spatial distribution of a study object in a study area [43]. It takes spatial association measures as the core and explores spatial aggregation and autocorrelation by visualizing and analyzing the spatial distribution of a given object [44]. It usually includes global spatial autocorrelation analysis and local spatial autocorrelation analysis.

The global spatial autocorrelation analysis evaluates the overall trend of spatial correlation throughout the whole research region using the following equation:

(11) $$\mathrm{Moran}’s\;I=\frac{\sum_{i=1}^{n}\sum_{j=1}^{n}W_{ij}{(A}_{i}-\bar{A}){(A}_{j}-\bar{A})}{S^{2}\sum_{i=1}^{n}\sum_{j=1}^{n}W_{ij}}\;$$

(12) $$S=\frac{1}{n}\overset{n}{\underset{i=1}{\sum }}{{(A}_{i}-\bar{A})}^{2}$$

In Equations (11) and (12), *n* is the number of study regions, *i* and *j* denote region *i* and region *j*, $A_{i}$ is the disaster resilience value of region *I*, and $w_{ij}$ is the spatial weight matrix. If Moran’s I value is greater than 0, it means that there is a positive correlation between disaster resilience and attributes between related counties; if it is less than 0, it is a negative correlation; if it is equal to 0, it is a random distribution, and there is no correlation.

Local spatial autocorrelation analysis represents the aggregation characteristics local to the analysis space. The calculated results are further characterized using local indicator spatial association (LISA) plots. There are four types: high-high (HH), high-low (HL), low-low (LL), and low-high (LH) [33].

#### 2.3.4. Partial Least Squares (PLS) Regression

The partial least squares (PLS) method combines the features of principal component analysis (PCA), typical correlation analysis (CCA), and linear regression analysis (LRA). The method can avoid the problems of bias and low sample size caused by autocorrelation among variables when applied to multiple linear regression models. It is one of the most effective methods to deal with multiple cointegration problems [45].

Linear regression can be used in identifying the degree of influence of each indicator on disaster resilience. This paper has 21 factors influencing disaster resilience, including five dimensions: environmental, economic, social, infrastructure, and disaster stress (see Table 1 and Section 2.3.6). However, there are certain correlations between the indicators of each dimension, and traditional regression analysis leads to large standard errors between the relevant independent variables.

Compared with traditional analysis, PLS can obtain acceptable bias estimates through bias–variance trade-offs with smaller mean squared errors in the independent variables. PLS can effectively address the problem of multicollinearity and support richer and deeper data, providing better results [46]. The equation is as follows:

(13) $${\mathrm{VIP}}_{j}=\sqrt{\frac{k}{{\mathrm{Rd}(y;t}_{1}{,\ldots t}_{m})}\overset{m}{\underset{h=1}{\sum }}{\mathrm{Rd}(y;t}_{h}{)W}_{\mathrm{hj}}^{2}}$$

considering the independent variable *X* and the dependent variable *Y*, and extracting latent variables *t_1_* and *u_1_* from *X* and *Y*, respectively. In Equation (13), ${\mathrm{VIP}}_{j}$ represents the *VIP* value of $x_{j}$; k is the number of independent variables, ${\mathrm{VIP}}_{1}^{2}{+\ldots +\mathrm{VIP}}_{p}^{2}=k$, $t_{1}{\ldots t}_{m}$ are principal components extracted from variable *X*, and ${\mathrm{Rd}(y;t}_{1}{,\ldots t}_{m})=\sum_{h=1}^{m}{\mathrm{Rd}(y;t}_{h})$ means the cumulative explanatory power of the primary components to *Y*. $W_{\mathrm{hj}}^{2}$ is the first j component of $w_{h}$-axis

#### 2.3.5. DROP Model

The paper defines resilience as the capacity of a system to utilize environmental, economic, social, and technological resources and other capital to resist and mitigate shocks in response to internal and external perturbations and transform society into a better state [13]. It has the characteristics of active adaptation and continuous change. Disaster resilience is the system’s ability to mitigate, contain, and reduce the impact of further disasters.

Researchers define disaster resilience as adaptive and inherent resilience (inherent resilience works during non-crisis times, adaptive resilience works during disasters) [37]. Both concepts are applied to infrastructures, institutions, organizations, social and economic systems, and organizational structures. In this paper, the disaster resilience of the place (DROP) model proposed by Cutter (2008) is used to evaluate inherent resilience, which is jointly determined by ecological, social, economic, and infrastructural variables [22]. In contrast to adaptive resilience, inherent resilience exists in the whole process of disaster and fluctuates with the changes in the structure and function of the system [47]. It is important to note that the use of inherent resilience can more effectively portray the impact of multi-hazards in Aba since the complete processes of disaster prevention, resistance, and rescue and the resilience of longer time series are considered in this paper. As a result, only the inherent resilience calculation is considered in this paper.

This paper collects publicly accessible online data to assess the resilience of the Aba in western Sichuan Province, as it is designed to integrate data from the previous ten years. In this process, available resilience research frameworks can be followed to obtain valid indicators that contribute positively or negatively to the resilience of the study area [48]. However, due to the uniqueness of the selected study region, it is unrealistic to follow a framework completely, and some adjustments should be made. It is reasonable to exclude indicators for which data cannot be queried to avoid the unavailability of indicator data affecting the study results. We used a portion of the DROP model, which initially comprised six dimensions: environmental, social, economic, infrastructure, community, and institutional, after combining the definition of resilience with the actual situation in Aba. The selected resilience assessment dimension is the entire system rather than the community level, making it challenging to define institutions and communities. In addition, Aba is affected by natural disasters, such as mudslides and landslides year-round; therefore, we added the disaster stress dimension to the evaluation model to account for multi-hazard impacts.

#### 2.3.6. Selection of Indicators

For the following reasons, this research establishes a framework of five dimensions: environment, economy, infrastructure, society, and disaster pressure.

A healthy natural environment is essential for risk reduction and resilience, and it can mitigate the damage caused by natural disasters. Economic characteristics represent the economic factors of preparedness and resilience in the face of disaster events. Financial capital can mitigate the damage caused by certain contingencies. Better social amenities within the county can make the region more robust to natural disasters. Infrastructure, which provides public services for social production and residential life, is the most crucial and fundamental component of urban resilience. Therefore, the region’s environmental, economic, social, and infrastructure conditions play a decisive role in natural disaster emergency preparedness and response capabilities [27]. It should be noted that the disaster pressure dimension represents the disaster potential points caused by the multi-hazard hazards faced by the study area selected in this paper. Establishing this dimensional index system can obtain the intuitive impact of disasters on the region and contribute to the improvement of the regional disaster preparedness and response capacity.

Based on the five dimensions of the framework, 21 indicators were selected for this paper.

(1) Environment. The environment is an important aspect of resilience assessment. This dimension includes forest coverage, average altitude, and per capita arable land. When natural disasters occur, the ecological environment undergoes structural changes, determining the speed of the city’s recovery from disasters. Better environmental conditions will be less affected by disasters or, to some extent, weaken the effects of disasters.

(2) Economy. Regions with strong economic and financial resources often correspond to faster recoveries and greater resilience [49]. The variables at the economic level include regional GDP per capita, the proportion of GDP of the tertiary industry, the total retail sales of social consumer goods per capita, local public finance revenue, and the balance of savings deposits per capita. Higher GDP reflects the improvement of a country’s national economic development, social security system, and infrastructure system, corresponding to higher disaster resilience. Higher fiscal revenue can pressure local governments’ disaster-resilience funds, better manage disaster risks, and thus improve resilience. The total retail sales of social consumer goods per capita reflect residents’ overall purchasing power in various regions. It reflects the basic living standards of residents to a certain extent. The higher the total amount, the more people have a more robust ability to resist and adapt to disasters [1]. Tourism is the leading industry in the study area and a critical pillar in stimulating local economic development. Tourism is an integral part of the tertiary industry, promoting the primary and secondary industries, and is an essential indicator for industrial upgrading and production optimization. A higher proportion of the tertiary industry represents a more robust regional economy.

(3) Society. The variables at the social level include the number of students in ordinary primary and secondary schools, the number of hospitals and social welfare beds per thousand people, the number of doctors per thousand people, the medical insurance rate, and the labor force ratio. According to existing research, obtaining higher medical insurance coverage and a more comprehensive education system are important measures to improve the overall resilience of the region, and medical security and social security reflect a more muscular primary social function [49].

(4) Infrastructure. Infrastructure, such as water and electricity lifelines, can help improve the well-being of regions after disasters, and upgrading critical facilities is essential for regional disaster resilience [50]. Therefore, the infrastructure level includes the number of fixed-line telephone users, the number of Internet users, the density of the road network, social electricity consumption, and the investment in fixed assets of society. More phone service and broader internet communications enable better responses during periods and preparedness for disasters. Longer road miles mean better planning for emergency evacuations [27].

In this paper, the indicator data were initially matriculated using Equation (3). The indicators were then normalized using Equations (4) and (5). Afterward, the indicator weights were computed using Equations (6)–(9). Finally, Equation (10) was utilized to obtain the indicator’s composite score. In addition, considering the whole process of disaster, the indicators of the four dimensions are organized according to the prevention, resistance, and rescue of disasters, as shown in Table 1.

## 3. Results

### 3.1. The Changing Trend of Disaster Resilience in Aba

**Figure 2:**
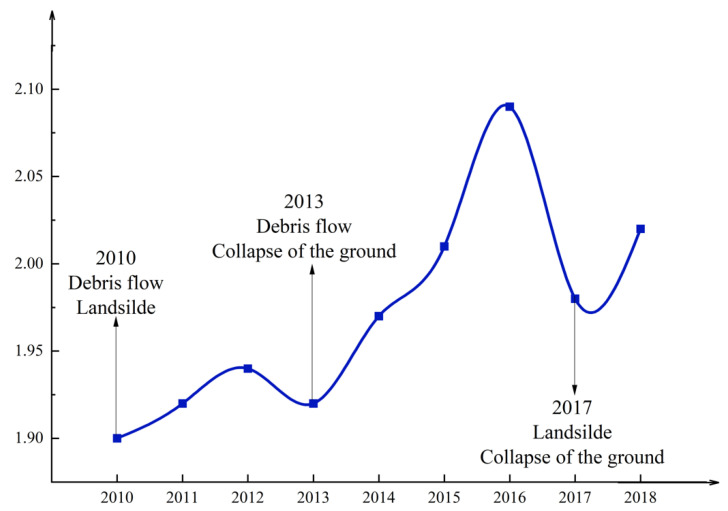
Changes in disaster resilience in Aba from 2010 to 2018.

According to the results of the calculations (Equations (1) and (2)), the overall disaster resilience change graph of Aba can be obtained, as shown in Figure 2. From Figure 2, it can be seen that the overall disaster resilience of Aba shows an upward trend, but the regional disaster resilience shows a downward trend in 2013 and 2017.

As depicted in Figure 2, Aba’s disaster resilience decreased in 2013 and 2017, which corresponds precisely to the years affected by severe natural disasters. In 2010, Wenchuan and Li were hit by debris flow and landslides, resulting in the blockage of many national highways, a disaster area of 10,000 square kilometers, more than 50,000 people affected, and direct economic losses of more than 600 million CNY. In 2013, Wenchuan broke out the ‘7.10’ debris flow and triggered the ground collapse and other disasters. The combined impact of multiple disasters caused 13 counties in Aba, 98 towns, and 175,000 people to be affected to varying degrees, with severe damage to road traffic, electricity, and communications, industry, agriculture, tourism, and water conservancy, resulting in direct economic losses of 6.786 billion CNY. In 2017, a large landslide occurred in Li, causing economic losses of 1.7 billion CNY. In the same year, a 7.0 magnitude earthquake occurred in Jiuzhaigou, triggering various secondary disasters, such as landslides and ground collapses, resulting in multiple new disaster potential sites in Ruoerge, Hongyuan, and Songpan. The direct economic loss exceeded 20 billion CNY. These severe natural disasters weakened Aba’s resilience and increased Aba’s vulnerability to natural disasters. Therefore, the curves in Figure 2 indicate that the indicator system used in this study can more accurately assess the trend of resilience in Aba under the influence of multi-hazards. Additionally, it indicates that the resilience framework can be used in highland mountainous areas.

In general, during 2010–2018, although the disaster resilience of the study area fluctuated to a certain extent, the threshold of the average disaster resilience continued to rise, and the overall trend showed a continuous increase. This phenomenon is closely related to the stable local ecological environment, the constant improvement of economic development, the improvement of social functions, the continuous improvement of the quality and quantity of infrastructure, and the continuous cleaning and investigation of multi-disaster hidden danger points.

**Figure 3:**
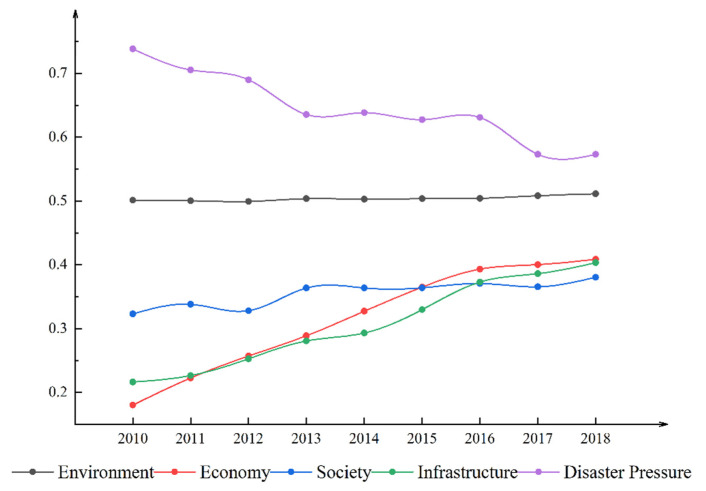
The quintuple structure of the disaster resilience in Aba.

The changing trend of the four components that make up the disaster resilience of Aba is shown in Figure 3.

It can be seen from Figure 3 that the environmental resilience of Aba is in a stable and rising state, which is related to the long-term stability of forest cover, elevation, and land use selected for assessing the environmental component. The resilience of the economy and infrastructure exhibited a yearly trend of sharp increases, which is inextricably linked to the Aba government’s increasing economic investment to achieve more rapid post-disaster recovery and reconstruction. In addition, it implemented numerous regional development strategies in pursuit of rapid economic growth. Over time, disaster stress rises (disaster stress is negatively treated, and smaller stress values represent more significant disaster stress), which is related to the vulnerability caused by the physical environment in which Aba is located. Compared with other components, the social resilience component also shows some increase during the study period, with the region’s social undertakings growing and livelihood security increasing. However, it exhibits considerable fluctuation, particularly around 2013 and 2017, when mega-disasters occurred. The combination of social resilience and disaster pressure offset the growth in the economic and infrastructure sectors, leading to a decrease in disaster resilience, as shown in Figure 2.

### 3.2. Temporal Variation of Disaster Resilience in 13 Counties

**Figure 4:**
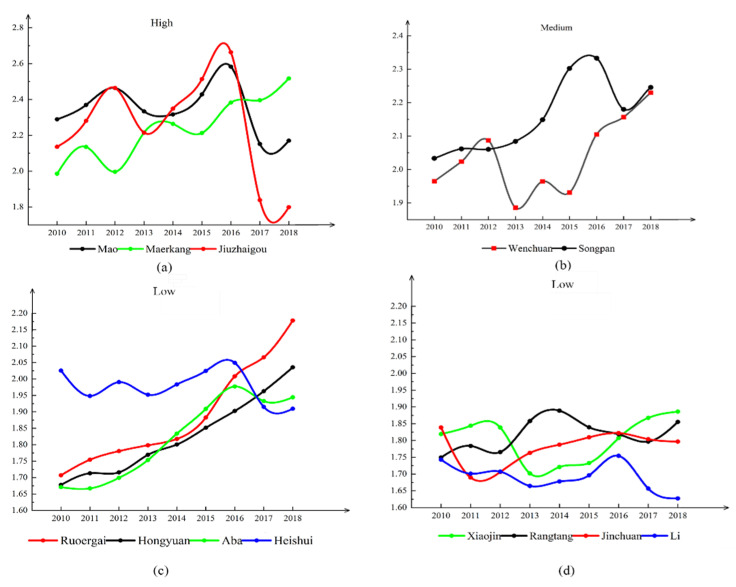
Changes in resilience in 13 areas. (**a**) high resilience regions; (**b**) medium resilience regions; (**c**,**d**) low resilience regions.

The resilience of 13 areas in Aba (2010–2018) under the influence of multi-hazards was calculated, using the resilience evaluation indicators constructed above (see Table 1). In this paper, the 13 regions of the Aba Prefecture are divided into three toughness grades: high, medium, and low, for trend mapping. The changing trend is shown in Figure 4.

From Figure 4, the level of disaster resilience shows a fluctuating increase, except for Li and Mao, thanks to the rising social, economic, and infrastructure levels, which offset the regional disaster pressure potential. Compared to other counties, the resilience of Mao, Maerkang, and Jiuzhaigou is at a high level (Figure 4a), and Wenchuan and Songpan are at a medium level (Figure 4b). The disaster resilience of Jiuzhaigou declined significantly after the multi-hazard impact in 2017, when its ability to withstand mitigation disasters took a hit. It should be noted that nearly all major disasters impacted Li (Figure 1) due to its geographic location. The potential pressure of disasters in the region exceeds the rise in social, economic, and infrastructure levels. After suffering from multiple disasters, resilience declines, making it more vulnerable and difficult to withstand and mitigate disaster shocks (Aba County exists in the Aba Prefecture, and all Aba in Figure 4 refer to Aba County).

**Figure 5:**
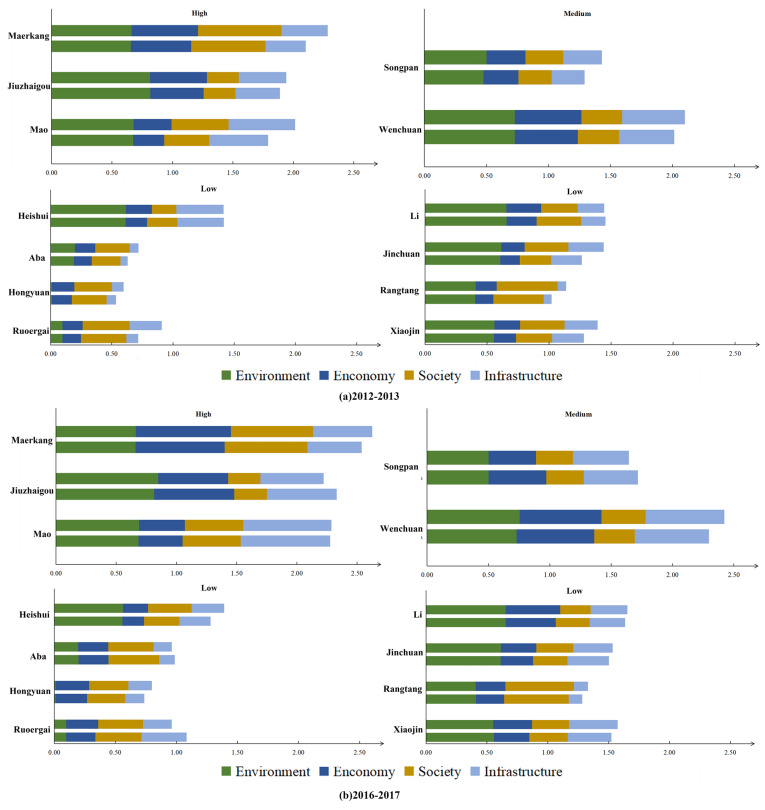
Changes in four dimensions of counties in Aba. (**a**). dimensional changes from 2012 to 2013; (**b**). dimensional changes from 2016 to 2017.

The area experienced natural disasters, such as landslides, debris flows, and earthquakes, with significant effects on all dimensions in 2013 and 2017. To visualize each county’s environmental, economic, social, and infrastructural changes. This paper shows a change graph in four dimensions for the region in the crucial years of 2013 and 2017. The trends of changes in 2012 and 2016 were also included for comparison purposes, as seen in Figure 5.

Figure 5 shows apparent gaps in each county’s development levels of the four dimensions. Moreover, the 2017 earthquake of magnitude 7.0 affected the economy and infrastructure of Songpan and Jiuzhaigou, causing them to decline relative to 2016 levels. It is worth mentioning that after the 2013 Wenchuan ‘710’ debris flow, the level of the four dimensions in Wenchuan increased somewhat compared to 2012 (Figure 4b), indicating that the area has some capacity to withstand and recover from the disaster. However, Wenchuan’s disaster resilience (Figure 4a) decreased relative to 2012, indicating that the 2013 disaster increased Wenchuan’s disaster stress and outweighed the benefits of the increased levels of the four dimensions.

This paper calculated coefficients of variation for the four components to explore the development gap between regions in Aba, as shown in Table 2. The coefficient of variation is determined by the standard deviation and mean of the data. A smaller coefficient of variation represents a minor degree of departure and vice versa. This paper uses the coefficient of variation to measure the balance of resilience development in the study area.

Environment, economy, society, and infrastructure were examined. The large values of the coefficient of variation for these four dimensions indicate that environmental, economic, social, and infrastructure levels between counties vary significantly. The variation coefficient of the social dimension increased from 27.1% in 2010 to 39.3% in 2018, indicating an increase in the disparity of social resources between countries. The coefficient of variation of the social resilience and environmental dimensions increased somewhat but generally stabilized. In contrast, the coefficient of variation of economic and infrastructure dimensions shows a decreasing trend, indicating that the Aba government keeps balancing economic development and infrastructure construction, which significantly reduces the imbalance of infrastructure and economic development between counties.

### 3.3. Spatial Variation in Disaster Resilience in 13 Counties

**Figure 6:**
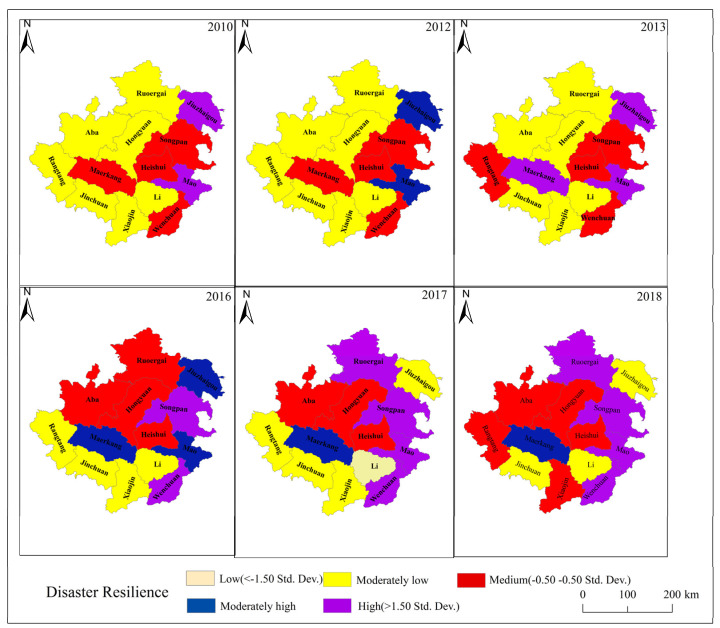
2010−2018 disaster resilience index of counties in the study area.

For data visualization and further research into the spatial clustering characteristics of changes in disaster resilience in each county, using the mean and standard deviation, this study categorizes the disaster resilience index of 13 areas in Aba from 2011 to 2019 into five levels: high, moderately high, medium, moderately low, and low, as shown in Figure 6 [51].

Regarding spatial distribution, disaster resilience is generally higher in the southeast and overall lower in the northwest, except for Maerkang. From 2010 to 2017, the disaster resilience in the eastern part of the study area was generally high. Unfortunately, after 2017, due to the impact of the Jiuzhaigou large-scale earthquake, the disaster resilience in the eastern part of the study area declined (Figure 5). To numerically visualize the spatial distribution of disaster resilience differences, global Moran’s I (Table 3) and local Moran’s I (Figure 6) can be calculated.

According to the global Moran’s I for disaster resilience in Aba, the Moran’s I is greater than 0 throughout the study period. The Moran’s I showed an upward trend in 2010–2016, but a decline in 2013, and the Moran index fell sharply in 2017–2018. In 2010–2016, the z-value was greater than the critical value (2.58), and the significant value level was 0.01, and in 2017–2018, the z-value was greater than the critical value (1.65), the significant value level was 0.1, and these results are significant. The results show a significant positive correlation between the distribution of disaster resilience in Aba and the disaster resilience of different counties, showing a significant spatial aggregation phenomenon.

It is worth noting that Moran’s I declined in 2013 but still showed a significant positive correlation across counties. The difference is that in the two years of 2017 and 2018, the n’s I of each county area are close to 0, which means that the degree of clustering of the disaster resilience distribution in the county area weakened, showing a random distribution trend.

**Figure 7:**
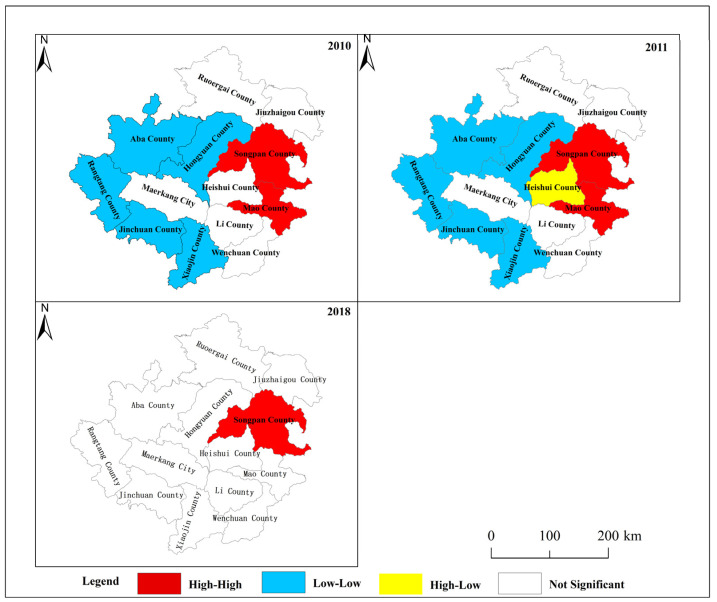
Disaster resilience LISA map of the Aba Prefecture from 2010 to 2018.

To further determine which counties in Aba have a spatial clustering phenomenon and analyze the spatial correlation of each county, this paper calculated local Moran’s I and plotted local indicators of spatial association (LISA), as shown in Figure 7.

HH, LL, and HL clusters existed in Aba during the study period. From 2010 to 2016, the HH clustering area was mainly in the eastern part of the Aba Prefecture, including Songpan and Mao, and the HL clustering area was mainly in Heishui, in the middle. In contrast, the LL-type counties are relatively widely distributed, and the western plateau counties in the Aba Prefecture show an LL-type distribution, indicating a strong local spatial positive correlation between the disaster resilience and the counties. After 2017, the disaster resilience of Aba changed greatly, and the spatial cluster of disaster resilience decreased. Only Songpan County has HH-type clusters.

### 3.4. Influencing Factors of Disaster Resilience

**Figure 8:**
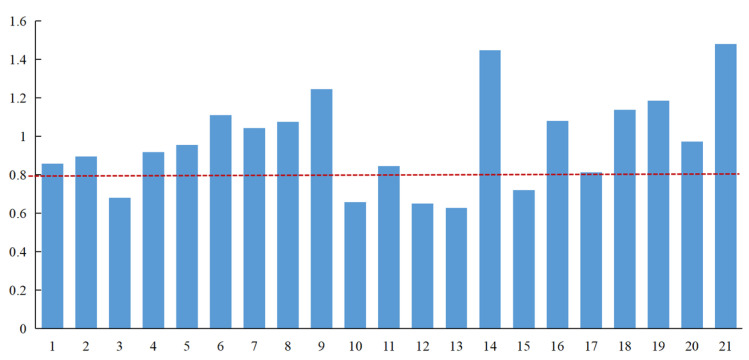
*VIP* values of factors affecting changes in disaster resilience.

Based on the PLS regression (see Section 2.3.4), the standardized regression coefficients of the factors influencing disaster resilience and the *VIP* values were calculated (see Table 4 and Figure 8), and the factors influencing the change in disaster resilience were analyzed (1–21 in Figure 7 correspond to the 21 indicators in Table 1, respectively).

From the results of PLS regression, 16 of the 21 indicators have *VIP* values greater than 0.8 (Figure 8). These indicators have a more significant impact on disaster resilience, where the indicators with the highest *VIP* values for each dimension were average elevation (*VIP* = 0.894), social consumer goods retail (*VIP* = 1.110), students (*VIP* = 1.244), communication devices (*VIP* = 1.447), and the collapse of the ground (*VIP* = 1.480).

From the regression coefficients, the coefficients of average elevation, residential savings, debris flow, landslide, and landslide hazards are negative, while the coefficients of the remaining indicators are all positive (Table 4). This indicates that, excluding these five indicators, all the remaining indicators show a positive effect on disaster resilience.

In summary, combining the regression coefficients and *VIP* values, it can be found that the key factors affecting resilience are average elevation, total social retail merchandise, fiscal revenue, number of doctors, communication equipment, social fixed asset investment, debris flow, landslide, and the collapse of the ground. Most notably, all three hazards of disaster stress show a strong negative impact on disaster resilience.

## 4. Discussion and Recommendations

### 4.1. Discussion

The paper discusses the results from environment, infrastructure, economy, society, disaster pressure, and spatial heterogeneity.

(1) Environment. County environmental levels remained relatively stable in 2013 and 2017, when they suffered from large disasters. However, the coefficient of variation between counties (see Table 2) is large and increased during 2010–2018, representing an increased imbalance in environmental aspects between counties. Among them, the three counties with the lowest rates are Hongyuan, Ruoergai, and Aba counties because the average altitude and the forest cover rate have the most significant effects on the environmental level. These three counties are situated in the northwest plateau region of the Aba Prefecture at a relatively higher altitude, have a relatively low amount of forest cover, and are experiencing grassland desertification. As a result, these regions are ecologically fragile and have a low carrying capacity for resources and the environment.

(2) Infrastructure. Hongyuan, Ruoerge, Aba, and Rangtang counties in Aba are the regions with the lowest infrastructure level (see Figure 5). These regions, which are in the transitional zone between high mountain valleys and plateaus, are impacted in their ability to develop economically by the difficulty of transportation and the slow adoption of communication and information technology.

(3) Economy. Because of its high concentration of ethnic minorities, remote location, and delicate ecological environment, the Aba Prefecture naturally has low socioeconomic development. At the same time, the county has imbalances in finance, industrial structure, and consumption levels. Wenchuan, Jiuzhaigou, and Maerkang counties have significantly higher economic levels than other areas. This is because Wenchuan and Jiuzhaigou have the best location conditions in the state, near the economically developed Sichuan Basin, which can effectively drive the economy. In addition, Maerkang, the capital town of Aba, is located in the middle and has a more robust economic drive. Fortunately, between 2010 and 2018, the coefficient of variation for the economic dimension (see Table 2) fell by more than ten percentage points, indicating that the economic level gap between counties is gradually decreasing.

(4) Society. This dimension is the most balanced of the four. Except for the higher social level in Maerkang, the other regions are relatively balanced (see Figure 5). Due to its status as the capital town of Aba, the Prefecture invested more in medical resources, social security, and education resources, which provided more employment opportunities and a larger labor force, further improving the social level.

(5) Disaster stress. The 2013 Wenchuan mudslide outbreak triggered a ground collapse and other disasters, and the 2017 Jiuzhaigou 7.0 magnitude earthquake triggered a variety of secondary disasters, such as landslides and ground collapse. These disasters led to an increase in disaster stress in the counties and caused a decrease in their ability to resist and mitigate the impact when dealing with internal and external disturbances. Disaster resilience is reduced (see Figure 4), and relying on capital that utilizes the four dimensions is complex. For example, the overall levels of Li and Heshui counties in 2013 and Jiuzhaigou, Ruoerge, Songpan, and Mao counties in 2017 decreased compared to the previous year (see Figure 7).

(6) Spatial Heterogeneity. The county’s environmental, economic, social, infrastructural, and disaster pressures led to substantial spatial heterogeneity in disaster resilience (see Figure 6). The western region of Aba is a high mountain fringe with higher elevation, making it more prone to geological hazards, such as landslides, landslides, and mudslides. At the same time, the environmental, economic, social, and infrastructure levels are lower than those in the eastern region, which leads to higher disaster resilience in the east region of Aba and relatively lower resilience in the western region. Therefore, there is a spatial aggregation phenomenon of HH aggregation in the east of the disaster resilience and LL aggregation in the west (see Figure 7). However, the earthquake in Jiuzhaigou in 2017 severely affected the eastern part of Aba. The pattern of disaster resilience in Aba changed, and the spatial aggregation effect decreased, showing a trend of random distribution.

Combining the above findings, it is uncomplicated to conclude that environment, infrastructure, economy, society, and disaster stress all together affect disaster resilience. Regions with lower levels of development and higher disaster stress tend to be more vulnerable and have lower disaster resilience (see Figure 4 and Figure 5). In other words, low disaster resilience frequently coexists with high vulnerability. When a region has low disaster resilience, the impact of disasters on the environment, infrastructure, economic, and social development levels will be more severe. The development levels determine disaster resilience, and disaster resilience in turn influences the development levels. This demonstrates that there is an interaction between disaster resilience and regional development.

Furthermore, the conclusions reached in this paper align with how Aba and its regions developed, indicating that the framework it presents for analyzing regional resilience is effective.

### 4.2. Policy Recommendations

Presently, Aba is facing frequent natural disasters and regional differences in development levels. Coping with the impact of multi-hazards and achieving sustainable and synergistic regional development became a vital future issue for Aba’s government. Based on the previous identification of important influencing factors, spatial analysis, changes in the four dimensions (see Figure 7) and gaps (see Table 2), and changes in disaster stress (see Figure 2), the following policy recommendations are proposed to promote economic development in Aba and reduce the differences in development levels between regions.

(1) Promote regional co-development. The large disparity in development levels among Aba’s counties (see Table 2) and areas with low disaster resilience clustering (see Figure 7) necessitate the need to balance regional development. Regional variations in development levels are unavoidable in Aba because of the unequal distribution of resources. The government of Aba ought to make use of the location. Wenchuan and Jiuzhaigou in the eastern region should strengthen cooperation with the neighboring areas to lead the economic development of the Aba Prefecture and achieve shared regional development. Wenchuan and Jiuzhaigou in the east of the region should enhance collaboration with the adjacent areas to lead the development of Aba and achieve shared regional development. Jinchuan and Heshui in the western region actively promote a resource-based economy, build high-quality tourist areas, quicken the growth of tertiary industry, and close the development gap between the eastern and western regions.

(2) Strengthening development level building. The capacity of a region to prevent, resist, and rescue from disasters, is determined by its environment, economy, society, and infrastructure. That is, a lower level of development represents greater vulnerability, and a higher level of development represents greater disaster resilience. Thus, improving these elements and the interconnection of the four dimensions leads to higher disaster resilience and lower vulnerability. Economically, the government of Aba should take advantage of the economies of Wenchuan, Jiuzhaigou, and Maerkang to stimulate social consumption within the region, boost social demand, and promote inter-regional linkages to achieve region-wide economic development. Environmentally, the government should promote urban and rural green construction, strengthen afforestation and compulsory tree planting efforts, and maintain soil and water to enhance forest coverage. Socially, the government should increase the ability to defend social organizations, expand the health sector’s operations, and safeguard health care services. Infrastructure-wise, the government should boost investment in infrastructure building, modernize communication equipment, public transit, etc., especially in the western regions such as Hongyuan, Ruoerge, Aba, and Rangtang.

(3) Emphasis is placed on catastrophe management and prevention. The growth in disaster pressure from 2010 to 2018 (see Figure 3) shows that each county’s potential disaster locations increased. Disasters, including earthquakes, landslides, and debris flows, are frequent in this area due to Aba’s special geographic location (see Figure 2). Multi-disasters significantly impact the development of disaster resilience, especially in the western region where disaster resilience LL is concentrated (see Figure 7). The government of Aba needs to focus on disaster prevention, resistance, and rescue efforts, regularly identify potential disaster sites, and develop strategies for avoidance and relocation to minimize the impact of the catastrophe.

The recommendations presented in this paper can help to promote the economic development of Aba, reduce the differences in development levels between regions, and improve disaster resilience to achieve sustainable regional development.

## 5. Conclusions

The paper used the DROP model’s four dimensions—environmental, economic, social, and infrastructure. Additionally, the disaster stress dimension was included to account for the effect of multi-hazards. Twenty-one indicators were selected to quantitatively assess disaster resilience in Aba during 2010–2018, analyze regional development differences and spatial and temporal changes in disaster resilience in Aba, and identify key influencing factors. The results show that the model can accurately assess how disaster resilience changed over time. The study discovered that each county in Aba has a different disaster resilience and development level for each dimension. Additionally, there is a spatial aggregation phenomenon where disaster resilience is H-H in the east and L-L in the west. To improve regional resilience to seek sustainable development in ecologically vulnerable areas, this paper proposes: (1) Promote regional co-development, and improve the level of regional development. (2) Strengthen economic and environmental dimensions, enhancing connections across dimensions. (3) Pay attention to disaster prevention and management, and establish a complete disaster prevention, resistance, and rescue system.

The paper improves the current calculation model, incorporates per capita disaster hazard potential points for disaster resilience calculation, and adds disaster pressure to the traditional resilience evaluation. It also chooses poor mountainous areas, currently understudied, for the study. This paper applies the regional resilience framework to the highland mountainous regions, which can serve as an example for other regional resilience studies similarly situated and impacted by multi-hazards. Furthermore, we apply the framework to China, a developing country, which serves as a reference for other countries facing similar challenges and multi-hazard disasters. However, this paper has some limitations, and future research may combine subjective and objective factors instead of leaving out subjective factors from the resilience calculation. In addition, expert scoring or other subjective weighting techniques may be used in conjunction with the calculation of each indicator’s weight. Moreover, only the inherent resilience under the influence of multi-hazards was calculated in this study, and the combination of adaptive resilience with inherent resilience can be considered.

## Figures and Tables

**Table 1:** Multi-hazard disaster resilience evaluation indicators.

	Dimension	Variable	Variable Description	Category	Weight
$f_{r}$	Environment	Coverage	Forest coverage (%)	Prevention	5.41%
		Elevation	Average elevation (m)	Prevention	7.01%
		Land area	Arable land area per capita (ha/person)	Resistance	3.04%
	Economy	GDP	Local GDP per capita (CNY)	Prevention	5.46%
		Industry structure	The proportion of tertiary industry in total GDP (%)	Prevention	5.57%
		Social consumer goods retail	Total retail sales of consumer goods per capita (CNY)	Prevention	5.98%
		Finance revenue	Local public finance revenue (ten thousand CNY)	Prevention	8.17%
		Savings	Residents’ savings per capita (CNY)	Prevention	5.28%
	Society	Students	Number of students on campus	Prevention	4.02%
		Bed space	Number of beds in hospitals and health institutions per 1000 population/unit	Rescue	6.26%
		The doctor	Number of physicians per 1000 population	Rescue	6.33%
		Social labor	Employ labor (%)	Resistance, Rescue	2.31%
		Social Security	Population with health insurance (%)	Resistance	2.23%
	Infrastructure	Communication Equipment	Number of fixed phone users	Resistance, Rescue	6.74%
		Public transport	Road mileage (km/sq km)	Resistance, Rescue	5.41%
		Electricity	Electricity consumption in society (ten thousand kwh)	Resistance	7.43%
		Social investment	Amount of investment in fixed assets of the whole society (ten thousand CNY)	Prevention, Resistance	4.55%
		Internet users	Number of internet users	Prevention, Resistance, Rescue	8.79%
$f_{p}$	Disaster pressure	Debris flow	Hazardous spots of debris flow disaster per 10,000 people		18.2%
		Landslide	Hazardous spots of landslide disaster per 10,000 people		28.5%
		The collapse of the ground	Hazardous spots of the collapse of the ground per 10,000 people		53.3%

**Table 2:** Variation of coefficient of disaster resilience in Aba.

	Environment	Economy	Society	Infrastructure
2010	50.4%	55.7%	27.1%	60.0%
2011	50.6%	54.4%	33.4%	59.8%
2012	50.6%	53.2%	31.5%	58.1%
2013	50.1%	49.1%	34.7%	57.5%
2014	50.2%	49.5%	31.9%	56.7%
2015	50.2%	49.6%	35.2%	54.8%
2016	50.2%	45.5%	34.9%	52.5%
2017	51.0%	43.8%	37.5%	48.8%
2018	51.4%	44.4%	39.3%	51.9%

**Table 3:** Global Moran’s I result of the Aba disaster resilience.

	Moran’s I	Mean	SD	z-Value	*p*-Value
2010	0.256	−0.0833	0.0941	3.676	0.001
2011	0.270	−0.0803	0.0900	3.895	0.001
2012	0.291	−0.0911	0.0890	4.298	0.001
2013	0.228	−0.0873	0.0920	3.424	0.001
2014	0.246	−0.0800	0.0942	3.460	0.001
2015	0.296	−0.0833	0.0909	4.245	0.001
2016	0.292	−0.0830	0.0907	4.131	0.001
2017	0.075	−0.0808	0.0925	1.685	0.057
2018	0.045	−0.0863	0.167	1.660	0.070

**Table 4:** PLS regression results.

Dimension	Variable	Coefficient	*VIP*
Environment	Coverage	0.222	0.856
	Elevation	−0.169	0.894
	Land area	0.059	0.679
Economy	GDP	0.003	0.918
	Industry structure	0.040	0.955
	Social consumer goods retail	0.171	1.110
	Finance revenue	0.225	1.041
	Savings	−0.127	1.075
Society	Students	0.023	1.244
	Bed space	0.169	0.657
	The doctor	0.019	0.844
	Social labor	0.211	0.650
	Social security	0.064	0.628
Infrastructure	Communication devices	0.411	1.447
	Public transport	0.229	0.720
	Electricity	0.189	1.080
	Social investment	0.316	0.812
	Internet users	0.123	1.138
Disaster pressure	Debris flow	−0.539	1.184
	Landslide	−0.238	0.973
	The collapse of the ground	−0.530	1.480

## Data Availability

Data sharing not applicable.
